# Acute Effects of Barbell Bouncing and External Cueing on Power Output in Bench Press Throw in Resistance-Trained Men

**DOI:** 10.3389/fphys.2022.899078

**Published:** 2022-06-06

**Authors:** Atle Hole Saeterbakken, Jorund Loken, Tom Erik Jorung Solstad, Nicolay Stien, Olaf Prieske, Suzanne Scott, Vidar Andersen

**Affiliations:** ^1^ Department of Sport, Food, and Natural Sciences, Faculty of Education, Arts, and Sports, Western Norway University of Applied Sciences, Bergen, Norway; ^2^ Division of Exercise and Movement, University of Applied Sciences for Sports and Management Potsdam, Potsdam, Germany; ^3^ Department of Sport and Health Sciences, University of Exeter, Exeter, United Kingdom

**Keywords:** stretch-shortening cycle, descending velocity, upper limb power, kinematic, force–velocity relationship

## Abstract

The aims of this study were to compare power output during a bench press throw (BPT) executed with (BPT_bounce_) and without (BPT) the barbell bounce technique, and examine the effect of cueing different barbell descent velocities on BPT power output in resistance-trained males. In total, 27 males (age 23.1 ± 2.1 years; body mass 79.4 ± 7.4 kg; height 178.8 ± 5.5 cm; and 4.6 ± 1.9 years of resistance training experience) were recruited and attended one familiarization session and two experimental sessions (EXP 1 and EXP 2). The force–velocity profile during maximal BPT and BPT_bounce_ (randomized order) under different loads (30–60 kg) was established (EXP 1), and the effect of varying external barbell descent velocity cues “slow, medium, and as fast as possible” (i.e., “fast”) on the power output for each technique (BPT and BPT_bounce_) was examined (EXP 2). Comparing two BPT techniques (EXP 1), BPT_bounce_ demonstrated 7.9–14.1% greater average power (*p* ≤ 0.001, ES = 0.48–0.90), 6.5–12.1% greater average velocity (*p* ≤ 0.001, ES = 0.48–0.91), and 11.9–31.3% shorter time to peak power (*p* ≤ 0.001–0.05, ES = 0.33–0.83) across the loads 30–60 kg than BPT. The cueing condition “fast” (EXP 2) resulted in greater power outcomes for both BPT and BPT_bounce_ than “slow.” No statistically significant differences in any of the power outcomes were observed between “medium” and “slow” cuing conditions for BPT (*p* = 0.097–1.000), whereas BPT_bounce_ demonstrated increased average power and velocity under the “medium” cuing condition, compared to “slow” (*p* = 0.006–0.007, ES = 0.25–0.28). No statistically significant differences were observed in barbell throw height comparing BPT and BPT_bounce_ under each cuing condition (*p* = 0.225–1.000). Overall, results indicate that both bouncing the barbell and emphasizing barbell descent velocity be considered to improve upper body power in athlete and non-athlete resistance-training programs.

## Introduction

Barbell bench press is one of the most frequently used resistance exercises for developing upper body strength and mechanical power (van den Tillaar, 2004; Bragazzi et al., 2020; Sakamoto and Sinclair, 2012; Cormie et al., 2011), particularly in sports involving explosive upper limb actions (e.g., throwing and striking). These movements require high velocity rather than high maximal strength in the upper body, as the ability of muscles to produce force decreases with increasing movement velocity (Young, 2006; Cormie et al., 2010). Establishing the force–velocity profile for a specific exercise enables the highest mechanical power output and the intensity (i.e., load and velocity) at which it is produced to be characterized on an individual basis (Wilson et al., 1993; Cronin et al., 2001a; Samozino et al., 2012; Jaric, 2015). Depending on the exercise type, equipment used, training status, and muscle groups elicited, power output is shown to be the greatest at intensities ranging between 30–70% of one repetition maximum (RM) (Wilson et al., 1993; Cronin et al., 2001b; Sakamoto et al., 2018; Đurić et al., 2021).

The traditional bench press technique adopted during power training is characterized by a large acceleration at the beginning of the barbell lift (ascent phase) (Newton et al., 1997; Baker and Newton, 2005; Tillaar and Ettema, 2013). However, high force generation, which produces barbell acceleration, is only observed during a small part of the ascent phase and is followed by a deceleration phase at the end of the barbell lift (Elliott et al., 1989; van den Tillaar and Ettema, 2009; van den Tillaar and Ettema, 2010; Pérez-Castilla et al., 2020). Furthermore, the deceleration phase is accompanied by a reduction in agonist muscle activity (Elliott et al., 1989; Newton et al., 1996; Sakamoto and Sinclair, 2012), which suggests that the traditional barbell technique may not provide the best approach to train maximal neuromuscular adaptions. In order to overcome the delimited reduction in active force production at the end of the ascent phase, the effect of implementing ballistic actions (e.g., projecting the barbell) has been studied (Newton et al., 1996; McEvoy and Newton, 1998; Sakamoto and Sinclair, 2012; Sakamoto et al., 2018; Pestaña-Melero et al., 2020; Løken et al., 2021). For example, Newton et al. (Newton et al., 1996) demonstrated that using a bench press throw technique (BPT) resulted in barbell acceleration during 96% of the ascent phase, compared to 60% using a traditional, non-ballistic bench press action. Furthermore, at intensities of 30–60% of 1-RM, greater peak angular velocity at the elbow (Sakamoto et al., 2018), higher peak and mean barbell velocity (Newton et al., 1996; Cronin et al., 2001a; Pestaña-Melero et al., 2020), and greater mean force and peak power (Newton et al., 1996; Pestaña-Melero et al., 2020) have been demonstrated for BPT than for non-ballistic, traditional bench press.

Typically, explosive actions exploit stored elastic strain energy and enhanced neural drive to agonist muscles derived from the stretch-shortening cycle (SCC) (Komi, 1984; Fukutani et al., 2020). Performance-enhancing effects of SCC typically result from an eccentric action (e.g., barbell descending phase) immediately preceding an explosive action (e.g., dynamic barbell ascent), as observed during throwing, jumping, or ball striking (Morriss and Bartlett, 1996; McMaster et al., 2014). In the context of bench press, concentric-only actions (i.e., barbell lifting) have been compared with actions involving both eccentric and concentric phases (e.g., barbell lowering followed immediately by barbell lifting) at different intensities (15–100%) of 1-RM, with greater velocity, acceleration, force, and power output reported for the eccentric-concentric action (Newton et al., 1997; Cronin et al., 2001b; Pérez-Castilla et al., 2020). These findings are in accordance with the generally agreed principle of implementing SSC components in training regimens aiming to increase velocity and power (Wilson et al., 1993; Newton et al., 1997; Cronin et al., 2001b; Boffey et al., 2019). However, the potential for barbell velocity during the lowering (i.e., eccentric) phase to affect power output during bench press has not been conclusively demonstrated. For example, Pryor et al. (Pryor et al., 2011) compared the effect of different lowering velocities at 80% of 1-RM during bench press lifting and demonstrated that higher barbell descending velocity (1 s descent phase) resulted in greater peak and average power output during the lifting phase, compared with a lower velocity (4 s descent phase). Carzoli et al. (Carzoli et al., 2019) demonstrated an increase in peak lifting velocity after a higher velocity descent phase, compared with the usual barbell descent cadence, at both 60 and 80% of bench press 1-RM. In experienced, bench press-trained participants, a fast-eccentric bench press action resulted in the greater mean and peak concentric barbell velocity, compared to a concentric-only action, but was similar to a controlled-eccentric action (1.5 s) under light and medium loads (30- and 50% of 1-RM) (Janicijevic et al., 2020). However, none of the studies cited implemented the bounce technique (BPT_bounce_) or the ballistic BPT during the bench press action (Pryor et al., 2011; Carzoli et al., 2019; Janicijevic et al., 2020).

Traditionally, it is recommended that the barbell should only lightly touch the chest and not rebound (i.e., bounce) off it (Løken et al., 2021). Theoretically, the bounce bench press may enable greater acceleration of the barbell than traditional approaches to the bench press technique, increasing power output during the exercise, particularly in the early part of the lift (ascent phase). However, Loken et al. (Løken et al., 2021) compared the training effects of bench press, with or without bouncing the barbell, in amateur handball players and found no difference in throwing velocity, 1-RM strength, or power output between the two approaches. Elsewhere, Krajewski et al. (Krajewski et al., 2019) compared a conventional deadlift, performed with and without bouncing the barbell, and demonstrated increased acceleration during the first 0.1 s of the lifting phase for the bounce technique. However, the effect of varying barbell lowering velocity with BTP_bounce_ during bench press throw (BPT) has not been explored.

Therefore, the present study aimed to characterize the acute effects of performing BPT with and without the bounce technique on mechanical power and barbell kinematics, and second, to examine whether externally cueing lowering velocity had an impact on power output in resistance-trained males. Based on the findings from previous studies (Newton et al., 1997; Cronin et al., 2001b; Pryor et al., 2011; Pérez-Castilla et al., 2020), we hypothesized greater power output for BPT_bounce_ than BPT, and that higher velocity during the barbell descent phase would increase the power outcomes.

## Materials and Methods

### Participants

With reference to Loken et al. (Løken et al., 2021) and with *α* = 0.05 and *β* = 0.80, the sample size of 24 subjects appeared to be necessary to detect significant differences in mean power between BPT bounce and BPT. In total, 27resistance-trained men (age 23.1 ± 2.1 years, body mass 79.4 ± 7.4 kg, height 178.8 ± 5.5 cm, and 4.6 ± 1.9 years of resistance training experience) were recruited. To be included, participants had to be free of injury, pain-free during maximal lifting, performing bench press as part of their weekly training routine, and with a 1-RM bench press of at least their own body weight. Participants were informed verbally and in writing regarding the implications and potential side effects of participating in the experiment, and were asked to refrain from any strenuous activity 48 h prior to testing. The study was conducted in accordance with the Declaration of Helsinki and confirmed by the Norwegian Centre for Research Data (ref. 288211).

### Study Design

The study used a within-subjects cross-sectional design. Participants visited the test location three times (one familiarization and two experimental visits: EXP1 and EXP2). The bench press throw (BPT) was conducted in a Smith machine (Pivot 680L, Pivot Fitness, Tianjin, China). In the familiarization session, BPT and BPT_bounce_ techniques were performed across a range of loads (20, 30, 40, 50, 60, and 70 kg) to ensure correct BP lifting and bouncing techniques were used. Each participant was given two to three attempts at each load for both BPT techniques. In EXP1, subjects performed maximal effort BPT, using both techniques in a randomized order, with loads ranging from 30 to 60 kg. All participants achieved peak power for loads in the 30–60 kg range. In EXP2, participants performed BPT and BPT_bounce_ under three externally cued lowering velocity conditions: “slow,” “medium,” and “as fast as possible” (i.e., “fast”), using the loads established in EXP1 corresponding to individual peak power output for each BPT technique. In addition, participants’ 1-RM for bench press (BP) was measured using the traditional BP technique.

### Procedures

Participants attended the lab three times over a period of 2 weeks; each visit was separated by 4–5 days. In the first session, participants were familiarized with BPT performed with and without the bounce technique. Session two examined the participants’ force–velocity profile across a range of loads (EXP 1) and session 3 (EXP 2) investigated the effect of different barbell lowering cues on BPT, with and without barbell bounce. Before entering the lab, participants completed a 5-min general warm-up (jogging or cycling). The warm-up continued in the lab with dynamic stretches for the pectoralis, anterior deltoid, and triceps brachii muscles, followed by 10 B P repetitions at 20kg, four repetitions at 50% of self-reported 1-RM, and two repetitions at 75% of self-reported 1-RM. Participants used their preferred grip- and feet-width, which were measured initially and then controlled before each subsequent lift in all sessions (Saeterbakken et al., 2011).

Familiarization with the BPT and BPT_bounce_ involved completing two to three trials (loading range: 20–70 kg) to lift the barbell using each technique. In BPT trials, participants were instructed to lower the barbell, lightly touch the chest (sternum position), and immediately press upward aiming for the maximal voluntary velocity of the barbell to the point of projection (i.e., barbell throw). Similar instructions were used for BPT_bounce_ trials, with the additional instruction to “bounce” the barbell off the sternum. For both techniques, participants were given the following instructions: “the aim is to lift the bar as fast as possible and lower the barbell fast, but with control.” For BPT, trials were rejected if the barbell bounced or if the lowering phase terminated at a visible distance (≥2 cm) above the chest. For BPT_bounce_, trials were rejected if the bar did not clearly make contact and then bounce off the chest. For both techniques, trials were rejected if the hips lifted off the bench, or if any hesitation occurred in the transition between the lowering and lifting phases.

In EXP1, power output for BPT and BPT_bounce_ was determined across the range of loads used. Typically, maximum power in BPT is produced with a load corresponding to approximately 50% of 1-RM (Baker et al., 2001; Sreckovic et al., 2015). Therefore, 30, 40, 50, and 60 kg loads were used to identify the load which elicited each participant’s average and peak power, average and peak velocity, and time to peak power and velocity. Previous studies examining BPT have demonstrated reliable measurement of power and velocity variables (within-participants coefficient of variation <5%, intra-class correlation coefficient >0.946) (García-Ramos et al., 2018a; García-Ramos et al., 2018b). Participants performed all lifts in a randomized order (i.e., either BPT or BPT_bounce_) under each loading condition, beginning with the lowest load. Immediate feedback on power output was used to motivate participants toward maximal effort. The average lower velocity was 0.99–1.04 m s^−1^ for BPT_bounce_ and 0.59–0.64 m s^−1^ for BPT. Rest between loads ranged from 1 to 3 min with 3 min rest between techniques. Three acceptable trials were performed at each load; however, only the trial with the highest average power (i.e., calculated from data gathered during the entire range of the ascending phase) was used in further analyses.

In EXP2, the effect on the power output of cueing three lowering velocities: “slow,” “medium,” and “fast” was examined for both BPT and BPT_bounce_. “Fast” corresponded to the same velocity achieved in the familiarization session and EXP1. A lift was not accepted if a participant increased the lowering velocity of the bar during the last part of the descent phase, that is, did not maintain a steady lowering velocity. In EXP 2, the load used corresponded to participants’ highest average power output for each BPT technique obtained in EXP1. The participants executed BPT and BPTb_ounce_ in a randomized order under each of the three lowering instructions.

After completing lifts using both techniques under all three lowering cues, a bench press 1-RM test was performed in the Smith machine at 90% of self-reported 1-RM, with 2.5–5.0 kg added stepwise, until the participant and test leader agreed that 1-RM was achieved. 1-RM was obtained within two to five attempts. A 5-min rest separated each trial.

To calculate power output, a linear encoder (Ergotest Innovation A/S, Porsgrunn, Norway) was attached to the barbell in both experimental sessions (EXP1; EXP2) to identify barbell peak velocity (pV), average velocity (aV), time to peak velocity (tpV), peak power (pP), average power (aP), time to peak power (tpP), and vertical displacement and velocity during the barbell lifting phase. The linear encoder had a resolution of 0.019 mm and a sampling rate of 200 Hz. Data were analyzed with the commercial software (Musclelab v.10.4.37.4073, Ergotest Innovation A/S, Porsgrunn, Norway). Unpublished data from the Norwegian School of Sport Sciences show that the encoder is reliable and valid for average velocity (*r* = 0.993, CV = 2.54) and displacement (*r* = 0.993, CV = 1.92) when compared with Qualisys Motion Capture Systems (Qualisys AB, Sweden).

In addition, the linear encoder was used to calculate barbell lowering distance (i.e., displacement from the start of the lowering phase to the point where the barbell touched the chest) for both BPT and BPT_bounce_ under each cueing condition. Accordingly, the lowering distance was subtracted from the ascending displacement to calculate the barbell throw height.

### Statistical Analysis

All baseline variables were tested for normality (Shapiro–Wilk test). Barbell lowering velocity and power output at each load were compared (i.e., BPT *versus* BPT_bounce_) using a paired *t*-test using SPSS statistical software (IBM Corp. Released 2020. IBM SPSS Statistics for Windows, Version 27.0. Armonk, NY: IBM Corp). To determine the effects of the three lowering cues, a two-way split-plot repeated analyses of variance (ANOVA) [within-subject factor: lowering cue (slow, medium, and fast)] x [between-subject factor: condition (BPT and BPT_bounce_)] was used. When differences were detected with ANOVA, paired t-tests with Bonferroni *post hoc* correction were applied. The magnitude of the effect was determined using Cohen’s d and interpreted according to the following scale: 0.0–0.2 (trivial), 0.2–0.5 (small), 0.5–0.8 (moderate), and >0.8 (large) (Komi, 1984). All data were reported as mean ± SD. The significance level was set to *p* < 0.05.

## Results

The participants’1-RM in bench press was 105 ± 16 kg corresponding to a relative strength (1-RM load/body weight) of 1.32. The load corresponding to the greatest average power out was 5.7% greater using the BPT_bounce_ compared to BPT (51.3 ± 11.3 kg vs. 48.5 ± 9.1kg, *p* = 0.022, ES = 0.27), and loads tested (30, 40, 50, and 60 kg) represented intensities of 29.1% (±3.9), 38.8% (±5.2), 48.5% (±6.5), and 58.3% (±7.9), respectively, of the participants’ bench press 1-RM. There were no differences in barbell lowering velocity across the loads for BPT_bounce_ (*p* = 0.666–0.901) or BPT (*p* = 0.280–0.622); however, the BPT lowering velocity was lower for all loads than BPT_bounce_ (*p* < 0.001–0.007).

### BPT_bounce_ vs. BPT (EXP 1)

**FIGURE 1:**
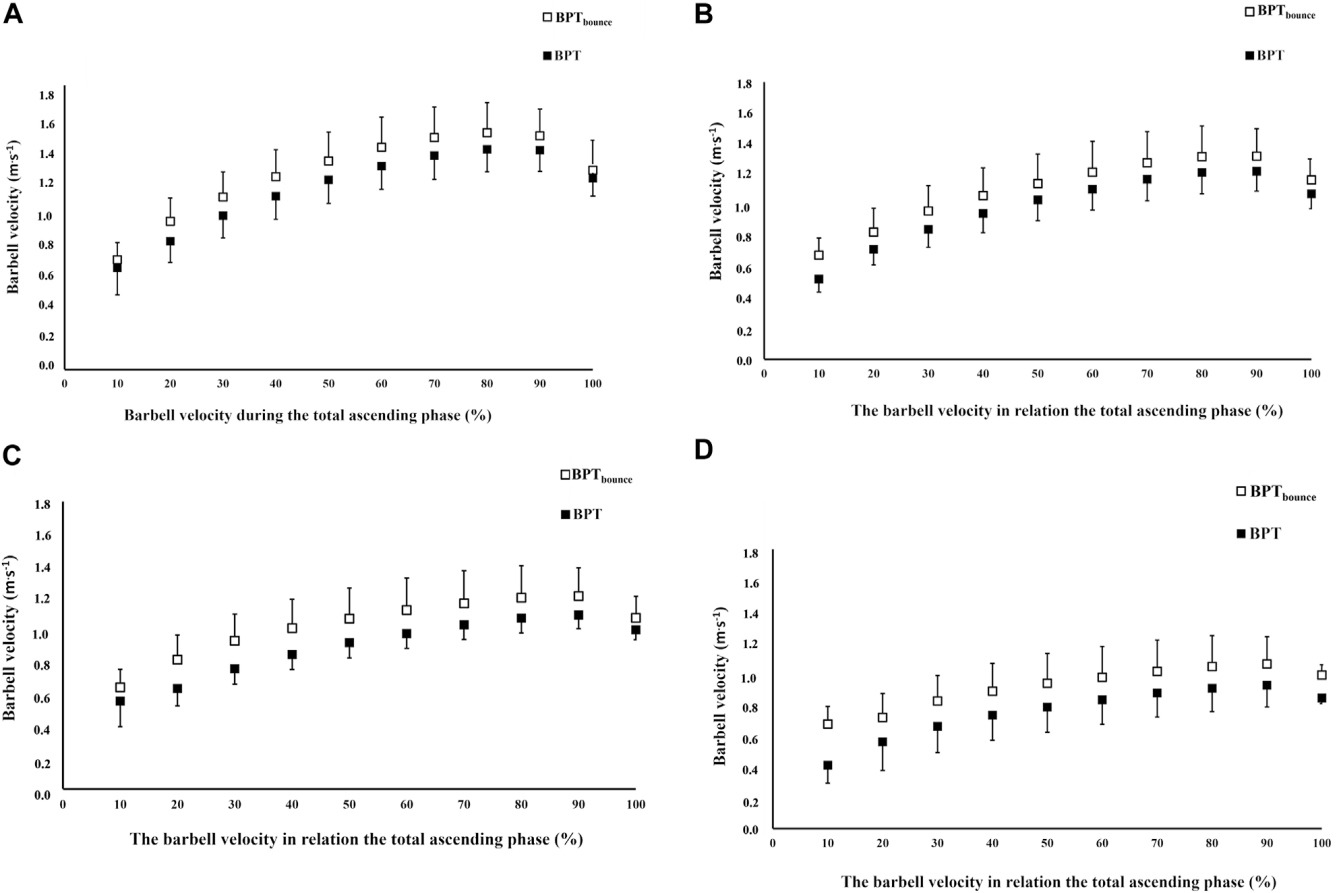
Barbell velocity relative to the barbell position to the ascending phase for the BPT_bounce_ technique and BPT for each 30 kg **(A)**, 40 kg **(B)**, 50 kg **(C)**, and 60 kg **(D)**.

Comparing the two BPT techniques, BPT_bounce_ demonstrated 7.8–14.1% greater average power (*p* ≤ 0.001, ES = 0.5–0.9), 6.5–12.1% greater average velocity (*p* ≤ 0.001, ES = 0.5–0.9), and 11.9–31.3% shorter time to peak power (*p* ≤ 0.001–0.05, ES = 0.3–0.8) across 30–60 kg than BPT (Table 1; Figure 1). BPT_bounce_ demonstrated 8.5–18.5% greater peak power than BPT for all loads (*p* = 0.003–0.007, ES = 0.4–0.7, Table 1), except for 30 kg (*p* = 0.369). For the variables’ peak velocity and time to peak velocity, no differences were observed between the two techniques at 30 and 60 kg (*p* = 0.057–0.875); however, BPT_bounce_ elicited 2.9 and 2.8% greater peak velocity and 4.7 and 7.9% shorter time to peak power at 40 kg (*p* ≤ 0.001, ES = 0.2–0.3) and 50 kg (*p* ≤ 0.001–0.011, ES = 0.1), respectively, than BPT (Table 1).

**TABLE 1:** Power output, velocity, and time in BPT with and without bounce.

Load (kg)	BPT technique	aP (w)	aV (m^.^s^−1^)	pP (w)	tpP (sec)	pV (m^.^s^−1^)	tpV (sec)
**30**	Bounce	488 ± 52^a^	1.31 ± 0.12^a^	927 ± 139	0.21 ± 0.07^a^	2.13 ± 0.20	0.28 ± 0.03
	No bounce	453 ± 44	1.23 ± 0.10	916 ± 129	0.23 ± 0.05	2.10 ± 0.17	0.28 ± 0.04
**40**	Bounce	572 ± 69^a^	1.18 ± 0.11^a^	1,026 ± 214^a^	0.20 ± 0.12^a^	1.83 ± 0.22^a^	0.32 ± 0.05^a^
	No bounce	512 ± 65	1.08 ± 0.11	945 ± 160	0.27 ± 0.07	1.77 ± 0.20	0.33 ± 0.05
**50**	Bounce	616 ± 93^a^	1.04 ± 0.13^a^	1,086 ± 294^a^	0.23 ± 0.15^a^	1.54 ± 0.23^a^	0.35 ± 0.06^a^
	No bounce	544 ± 78	0.94 ± 0.11	916 ± 167	0.33 ± 0.08	1.50 ± 0.20	0.38 ± 0.07
**60**	Bounce	606 ± 118^a^	0.88 ± 0.14^a^	988 ± 280^a^	0.33 ± 0.18^a^	1.30 ± 0.23	0.42 ± 0.12
	No bounce	530 ± 118	0.79 ± 0.15	873 ± 226	0.42 ± 0.13	1.26 ± 0.27	0.47 ± 0.12

a Significant difference between BPT techniques (*p* < 0.05).

aP, average power; aV, average velocity; pP, peak power; tpP, time to peak power; pV, peak velocity; tpV, time to peak velocity.

### Lowering Cues (EXP 2)

**FIGURE 2:**
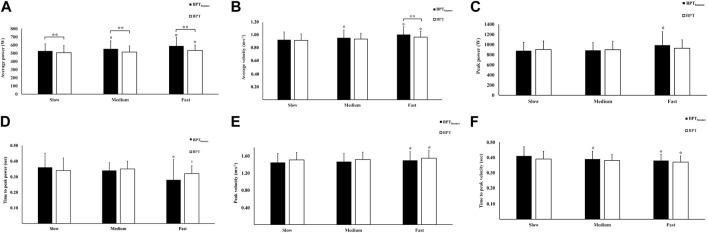
Effect of verbal cueing on bench press performance variables for the BPT_bounce_ technique and BPT on average power **(A)**, average velocity **(B)**, peak power **(C)**, time to peak power **(D)**, peak velocity **(E)**, and time to peak velocity **(F)**. ^∗^Significant difference compared to the other lowering cues. #Significant difference compared to “slow” lowering cue. †Significant difference compared to “medium” lowering cue. ^∗∗^Significant difference compared to BPT_bounce._

There was a significant interaction between condition and lowering cue for the following outcomes: average power (*F* = 5.574, *p* = 0.005), average velocity (*F* = 4.193, *p* = 0.020), and time to peak power (*F* = 3.307, *p* = 0.045), whereas for peak power, peak velocity, time to peak velocity, barbell lowering distance, and lowering velocity, no interaction (*F* = 0.304–3.058, *p* = 0.078–0.736) or main effect of condition (F = 0.037–1.441, *p* = 0.242–0.849) was observed, but there was a main effect for lowering cue (*F* = 197.623–8.465 *p* ≤ 0.001–0.003). For barbell throw height, no interaction (*F* = 2.101, *p* = 0.139) or main effect (F = 0.049–0.298, *p* = 0.716–0.827) was observed. All *post hoc* tests are presented in Figures 2A–F, Table 2, and Supplementary Tables S1, S2.

**TABLE 2:** Effects of externally cued lowering velocities on barbell kinematics.

Lowering cue	BPT technique	Ld (cm)	LV (m^.^s^−1^)	BPT height (cm)
Slow	Bounce	40.65 ± 5.40	0.34 ± 0.17	17.83 ± 3.74
	No bounce	37.80 ± 5.83	0.28 ± 0.12	16.33 ± 5.75
Medium	Bounce	39.98 ± 5.08	0.54 ± 0.16^b^	18.66 ± 3.40
	No bounce	37.50 ± 5.97	0.48 ± 0.15^b^	16.37 ± 6.23
Fast	Bounce	42.07 ± 5.15^a^	0.92 ± 0.18^a^	16.33 ± 5.75
	No bounce	38.49 ± 6.34	0.79 ± 0.17^a^	18.40 ± 4.06

a Significant difference compared to the other lowering cues.

b Significant difference compared to “slow.”

Ld, lowering displacement; LV, lowering velocity; BPT, bench press throw.

## Discussion

The aim of this study was to compare the effects of BPT_bounce_ with BPT and different external lowering cues on power outcomes. The main findings were that 1) BPT_bounce_ displayed greater average and peak power, and barbell velocity than BPT for the loads 40, 50, and 60 kg; 2) lowering the barbell “fast” demonstrated resulted in higher average and peak power, average and peak barbell velocity, independent of BPT technique, than “slow”; and 3) independent of lowering cue, BPT_bounce_ displayed greater average power than BPT.

In agreement with our hypothesis, whereas performance, characterized by higher output in all variables except average power and velocity, was greater for BPT_bounce_ at 40 and 50 kg, greater power and velocity were displayed at 30 and 50 kg. In the BPT_bounce_ technique, the barbell is brought down against the chest before being re-accelerated into the lifting phase. This action may improve the transition of energy from the descending to the ascending phase, resulting in greater acceleration and barbell velocity than the traditional BPT technique (Figure 1). The chest wall and its enveloping facia, which give the thorax its structural flexibility and contribute to respiratory mechanics (Smith et al., 2018), have the potential to be compressed, which may cause a “spring effect” as the barbell is bounced off the chest. Using a rebounding action (lowering + lifting) has been shown to elicit greater power output and barbell velocity rather than employing a lifting only action (Newton et al., 1997; Cronin et al., 2001b; García-Ramos et al., 2018b; Janicijevic et al., 2020; Pérez-Castilla et al., 2020; Pestaña-Melero et al., 2020). However, none of these studies included the barbell bounce technique, which could potentially utilize the SSC to derive performance gains to a greater extent than the rebounding-only action. To the best of our knowledge, only one previous study has examined acute effects of the bounce technique on force profile outcomes during deadlift at 75% of 1-RM (Krajewski et al., 2019). Krajewski and others (Krajewski et al., 2019) demonstrated that less force was required and lifting time was reduced, during both the initial lifting phase and throughout the barbell ascent. This study is not directly comparable with the present one in bench press however, as Krajewski and others (Krajewski et al., 2019) compared outcomes from five repetitions, under different loads, and for a different resistance exercise, that is, compound action. Furthermore, the mechanics of deformation (compression) and elastic recoil from an object striking the chest wall compared with the floor cannot be directly compared.

Of note is the finding that at 30 and 60 kg, no advantage was observed for the bounce technique on time to peak velocity or peak velocity, which could be related to the participants’ background in resistance training rather than athlete conditioning. Potentially, and according to the force–velocity relationship and training specificity principles (Behm and Sale, 1993), strong or powerful athletes demonstrate greater power output at either a higher or lower percentage of 1-RM (Cronin et al., 2000; Cronin and Sleivert, 2005; Loturco et al., 2019). However, none of the participants were athletes involved in throwing or striking, but were experienced in resistance training focusing on maximal strength and muscle hypertrophy (i.e., high force generation with relatively low barbell velocity). Probably, and as a result of their training background, the greatest average power output was achieved at 49% (51.3kg, BPT_bounce_) and 46% (48.5 kg, BPT) of 1-RM. This may explain why 30 kg did not demonstrate any advantage for the bounce technique for the outcomes of peak power, peak velocity, and time to peak velocity. Alternatively, lighter loads may result in less compression (i.e., deformation) of the thoracic cage and therefore reduce the potential advantage (i.e., spring effect) of the bounce technique. Of interest, Cronin et al. (Cronin et al., 2001b) examined power output in males with an athletic background across a range of loads (30–80% of 1-RM) and reported the greatest average power at 50% of 1-RM in BPT. It is also possible that the heaviest load (60 kg) may have caused participants to self-calibrate their output (e.g., reducing barbell velocity as it collides with the chest for reasons of safety), which could compromise the potential of the bounce technique to elicit high power output at increased loads.

The advantages of bouncing the barbell, compared to using the traditional technique, may be derived from its effect on lowering velocity (Janicijevic et al., 2020). As a direct consequence of bouncing the barbell, barbell lowering velocity was greater than that for BPT. In traditional bench press, lowering the barbell rapidly has been shown to result in greater average barbell velocity than lowering at a controlled pace (1.5 s descent phase) for loads ranging between 30–75% of 1-RM (Janicijevic et al., 2020). However, as Janicijevic and others (Janicijevic et al., 2020) did not examine either the BPT or BPT_bounce_ technique, we cannot infer from their results that differences in lowering velocity in our study caused the present findings.

In general, and supporting our hypotheses, cueing barbell descent velocity at different speeds using external verbal instruction had an impact on power outcomes, with the largest effect observed for comparison between “fast” and “slow,” which is not unexpected. Furthermore, for both BPT techniques, performance was superior (i.e., power indices increased) using the cue to lower “fast,” which supports the practice of using external verbal encouragement to enhance power outcomes during RT with the bench press. No difference was observed between the “medium” and “slow” velocity cueing conditions for BPT, a finding which could be of value in applied settings, as it suggests that lowering the bar more slowly may not result in power reduction, which could benefit less experienced practitioners, who may be technically less adept at throwing the bar, aiming to use this technique to enhance strength adaptations. Unlike for BPT, using BPT_bounce_, greater average power and velocity, and time to peak velocity were observed for the lowering cue “medium” compared to “slow.” This finding suggests that technical capacity should be sufficient to perform this variation in technique at a faster than controlled (e.g., 1.5 s descent phase) velocity, to access performance gains attributed to SSC-related mechanisms, as proposed here.

Theoretically, lowering the bar at a higher velocity could generate greater chest bounce (elastic recoil) in addition to eliciting greater stretch–reflex activation, increasing storage of energy in the tendons, promoting neurosensory pre-activation, and enhancing cross-bridge kinetics (Fukutani et al., 2020; Janicijevic et al., 2020). It should be noted that differences in velocity between the three cueing conditions were significant and lowering velocity increased by approximately 50% between each level of cueing (Table 2). Nevertheless, the present study found only limited evidence to support the speculation that the barbell bouncing technique exploits tissue biomechanical properties relating to the SSC which, if demonstrated, could offer a mechanistic explanation for findings elsewhere that BTP_bounce_ improves the power profile during BTP (Janicijevic et al., 2020). It is important to consider that at a higher barbell lowering velocity, greater force is required to decelerate the barbell, either to lightly touch the chest (BPT) or to strike against it and bounce off (BPT_bounce_). However, as none of the participants conducted bench press using the bounce technique regularly, it is possible that participants’ focus was directed towards the descending phase (barbell lowering) and not necessarily on the transition between movement phases. Therefore, lack of familiarization with technical aspects of actions examined (i.e., power training, bench press throw, and bouncing) and individual differences in responsiveness to external auditory cues (lowering instructions) could have influenced the results. Still, previous studies have demonstrated highly acceptable reliability for power and velocity outcomes in BPT, at similar loads and participants’ training status as the present study (García-Ramos et al., 2018a; García-Ramos et al., 2018b). Despite the potential limitation of only one familiarization session, the time to peak velocity was shorter under the “fast” cueing condition than that under the other velocity conditions, which is in agreement with our hypothesis. This could be explained by greater capacity to derive and then utilize SSC gains when the barbell was lowered at higher velocity, even though mean and peak velocity were similar across all lowering instructions.

Present findings are difficult to be compared with those of previous studies. For example, Pryor and others (Pryor et al., 2011) examined sets of bench press at 80% of 1-RM to fatigue and reported higher repetitions to failure, and greater average and peak power, for 1 *versus* 4 s lowering phase. In the present study, lowering time under the “slow” instruction was 1.25 s and under the “medium” instruction was 0.8 s, which are closer to, and less than the “fast” condition examined by Pryor et al. (Boffey et al., 2019). Elsewhere, in resistance-trained men, Carzolie et al. (Carzoli et al., 2019) examined the effect of bench press at 60 and 80% of 1-RM under two conditions: 0.75 (slow) and 2.0 (fast) times the individual’s normal lowering velocity, and found that both slow and fast lowering velocity resulted in greater peak and average ascending velocity than the participants’ normal velocity for the 60% of the 1-RM load (Carzoli et al., 2019). More recently, and supported by the findings of the present study (EXP 2), Janicijevic et al. (Janicijevic et al., 2020) demonstrated greater mean velocity for bench press at 30, 50, and 75% of the 1-RM load for fast, compared to controlled (duration of 1.5s), barbell lowering velocity. Compared with controlling lowering velocity, greater mean velocity was only reported under the heaviest load condition (75% of 1-RM), a finding which is comparable to results observed for the lowering cue “slow” in the present study.

Comparing BPT with BPT_bounce_, bouncing the barbell resulted in greater average power under all velocity cueing conditions. For BPT_bounce_, using the cue to lower the barbell “fast,” the average velocity was greater than that for BPT. For the other power outcomes, non-significant differences between BPT techniques and lowering cues were observed. This suggests lowering velocity is a more significant influence on power output than whether the bounce technique is included or not. Of note, a non-significant increase in barbell lowering displacement was observed using the bounce technique compared with BPT, which tends to confirm that participants produced a distinct bounce action, increasing the barbell’s path of movement by 2.5–3.5 cm (Table 2). A longer movement path, in addition to greater barbell acceleration in the early phase, may explain why average power was the only outcome variable that increased using the BPT_bounce_ technique compared with BPT, whereas peak power and other variables examined did not differ between techniques.

No difference in barbell throw height was found under any cueing condition, or comparing between the two BPT techniques. For both techniques, loads that elicited the greatest average power in the trial phase were used, which could explain why no significant differences in barbell throw height were found, as the load for BPT_bounce_ was 5.7% greater than that for BPT. Typically, the greatest benefits of the bouncing technique are evident in the early part of the barbell ascent phase (Krajewski et al., 2019), but may not necessarily translate into improvements in later parts of the lifting phase. In the terminal phase of the lift, barbell velocity increases (Saeterbakken et al., 2020; van den Tillaar and Saeterbakken, 2013), which influences the ability to apply high force at high velocities (Loturco et al., 2019). For example, Loturco et al. (Loturco et al., 2019) demonstrated greater power production among power-trained athletes in BPT than hypertrophy-trained athletes, which suggests that factors other than absolute strength, such as technique and timing, may influence force profile outcomes during the bench press. Previous studies have shown greater power output and velocity using the BPT than the traditional bench press technique (i.e., ending the barbell lift with fully extended elbows) (Newton et al., 1996; Cronin et al., 2001b). The proposal that greater lowering velocity enhances potential SSC gains remains debatable. For example, in the context of SSC in the lower limb, Ruffieux et al. (Ruffieux et al., 2020) demonstrated greater jump height with countermovement jump training than drop jump training among non-professional female volleyball players. Similarly, this finding has been reproduced at different drop jump heights (30–70 cm), although no difference in absolute jump height was demonstrated (Taube et al., 2012). Furthermore, Loken et al. (Løken et al., 2021) examined the effects of BPT_bounce_ compared with BPT (40–60% of 1-RM, three sets, three to five repetitions, twice per week) on throwing velocity, power output, and strength among handball players, and found no difference between the groups after 8 weeks. The authors speculated that the relative 1-RM strength level of the bounce group was too low to exploit potential gains from utilizing the bounce technique.

Even though the present study presents novel findings, some limitations need to be addressed. Loads corresponding to the greatest average power output (EXP1) were used to examine the impact of varying lowering velocity cues (EXP2). It is plausible that using other loads in EXP2, results might have been different, although we deliberately used mean power and not peak power to prescribe loads in EXP2. Several investigators have argued that peak power is a more reliable measure than mean power (García-Ramos et al., 2018a; Pestaña-Melero et al., 2020); however, none of these studies examined BPT_bounce_. Furthermore, although all participants were resistance-trained, they did not use the bounce technique in their regular training; therefore, familiarization with both a novel technique and external velocity cueing may have required more than the single session allocated. In addition, as the present study only included resistance-trained males, findings cannot be generalized to other populations. Small sample size, large variation between individual participants in training exposure and technical capacity, and conservative *post hoc* corrections may increase the risk of a type II error, when comparing the effect of varying lowering velocities on force profile outcomes. Of note, none of the participants experienced injuries as a result of this study, but some reported minor chest soreness from the bouncing technique.

## Conclusion

At loads of 30–60 kg, BPT_bounce_ elicited greater average power, average velocity, and time to peak power than BPT, and may therefore be superior, if high power output throughout the BP action is the desired outcome of prescribing BP training. Our findings suggest that if the bounce technique is preferred to throwing the barbell, technical proficiency should be sufficient to perform the descent phase action at a higher velocity, as power outputs were significantly greater at medium than controlled (1.5 s) descent velocity of the barbell for this technique. Overall, lowering the barbell at higher velocity increased power outputs across all variables, and seems to be of more importance than whether BPT with or without the bounce technique is adopted. In conclusion, while athletes involved in throwing-related sports may benefit from bouncing the barbell, irrespective of technique, emphasizing velocity during barbell descent is recommended to maximize power output.

## Data Availability

The raw data supporting the conclusion of this article will be made available by the authors, without undue reservation.
