# Supplementary material for: Acute Effects of Barbell Bouncing and External Cueing on Power Output in Bench Press Throw in Resistance-Trained Men
Source: Front Physiol. 2022 Jun 6;13:899078. doi: 10.3389/fphys.2022.899078 (PMC9208083; doi:10.3389/fphys.2022.899078)
Supplement: Supplementary file 1 [file Table1.docx]

Supplementing file 1. The effects of externally cued lowering velocities on BPT with bounce.

| **Lowering cues** | **Slow** | **Medium** | **Fast** |
| --- | --- | --- | --- |
| **aP (W)** | 529 ± 86 | 555 ± 98#, p=0.007, ES=0.3 | 591 ± 103*, p≤0.001–0.003, ES=0.4–0.7 |
| **aV (m^.^s^-1^)** | 0.93 ± 0.12 | 0.96 ± 0.12#, p=0.006, ES=0.3 | 1.01 ± 0.12*, p≤0.001–0.003, ES=0.4–0.7 |
| **pP (W)** | 881 ± 161 | 888 ± 152 | 990 ± 264#, p=0.036, ES=0.5 |
| **tpP (sec)** | 0.36 ± 0.09 | 0.34 ± 0.05 | 0.28 ± 0.13*, p=0.003–0.041, ES=0.6–0.7 |
| **pV (m^.^s^-1^)** | 1.45 ± 0.21 | 1.47 ± 0.19 | 1.50 ± 0.20#, p=0.001, ES=0.2 |
| **tpV (sec)** | 0.41 ± 0.06 | 0.39 ± 0.05#, p=0.003, ES=0.4 | 0.38 ± 0.04*, p≤0.001–0.005, ES=0.2–0.6 |
| **Ld (cm)** | 40.65 ± 5.40 | 39.98 ± 5.08 | 42.07 ± 5.15*, p≤0.001, ES=0.3–0.4 |
| **LV (m^.^s^-1^)** | 0.34 ± 0.17 | 0.54 ± 0.16#, p≤0.001, ES=1.2 | 0.92 ± 0.18*, p≤0.001, ES=2.2–3.3 |
| **BPT height (cm)** | 17.83 ± 3.74 | 18.66 ± 3.40 | 16.33 ± 5.75 |

* significant difference compared to the other lowering cues. # significant different compared to “slow” lowering cue. aP = average power, aV = average velocity, pP = peak power, tpP = time to peak power, pV = peak velocity, tpV = time to peak velocity, Ld = lowering displacement, LV = lowering velocity, BPT = bench press throw, ES = effect size.
