# Supplementary material for: Acute Effects of Barbell Bouncing and External Cueing on Power Output in Bench Press Throw in Resistance-Trained Men
Source: Front Physiol. 2022 Jun 6;13:899078. doi: 10.3389/fphys.2022.899078 (PMC9208083; doi:10.3389/fphys.2022.899078)
Supplement: Supplementary file 2 [file Table2.docx]

Supplementing file 2. The effects of externally cued lowering velocities on BPT without bounce.

| **Lowering cues** | **Slow** | **Medium** | **Fast** |
| --- | --- | --- | --- |
| **aP (W)** | 508 ± 86 | 516 ± 74 | 536±66*, p=0.001–0.002, ES=0.3–0.4 |
| **aV (m^.^s^-1^)** | 0.92 ± 0.10 | 0.94 ± 0.09 | 0.97 ± 0.09*, p<0.001, ES=0.3–0.5 |
| **pP (W)** | 905 ± 167 | 900 ± 168 | 929 ± 165 |
| **tpP (sec)** | 0.34 ± 0.08 | 0.35 ± 0.05 | 0.32 ± 0.05†, p=0.015, ES=0.6 |
| **pV (m^.^s^-1^)** | 1.51 ± 0.17 | 1.52 ± 0.17 | 1.55 ± 0.18*, p=0.020–0.050, ES=0.2 |
| **tpV (sec)** | 0.39 ± 0.05 | 0.38 ± 0.04 | 0.37 ± 0.04*, p=0.004–0.017, ES=0.3–0.4 |
| **Ld (cm)** | 37.80 ± 5.83 | 37.50 ± 5.97 | 38.49 ± 6.34 |
| **LV (m^.^s^-1^)** | 0.28 ± 0.12 | 0.48 ± 0.15#, p≤0.001, ES=1.5 | 0.79 ± 0.17*, p≤0.001, ES=1.9–3.5 |
| **BPT height (cm)** | 16.33 ± 5.75 | 16.37 ± 6.23 | 18.40 ± 4.06 |

* significant difference compared to the other lowering cues. # significant different compared to “slow” lowering cue. † significant difference compared to “medium” lowering cue. aP = average power, aV = average velocity, pP = peak power, tpP = time to peak power, pV = peak velocity, tpV = time to peak velocity, Ld = lowering displacement, LV = lowering velocity, BPT = bench press throw, ES = effect size.
